# Long-Term Kidney Outcomes in Paediatric Osteosarcoma Survivors: A 20-Year Multi-Centre Study

**DOI:** 10.3390/cancers18091391

**Published:** 2026-04-28

**Authors:** Christy Yuen-Kwan Mak, Dennis Tak-Loi Ku, Anthony Pak-Yin Liu, Vincent Lee, Jeffrey Ping-Wa Yau, Eric Chun-Ho Fu, Evelyn Ruoyun Lu, Matthew Ming-Kong Shing, Irene Yuk-Ying Ho, Will Wai-Lun Pak, Desmond Yat-Hin Yap, Alex Wing-Kwan Leung, Frankie Wai-Tsoi Cheng, Alison Lap-Tak Ma, Eugene Yu-Hin Chan

**Affiliations:** 1 Haematology and Oncology Centre, Department of Paediatrics and Adolescent Medicine, Hong Kong Children’s Hospital, Hong Kong SAR, China; christymyk@hku.hk (C.Y.-K.M.); anthony.liu@sickkids.ca (A.P.-Y.L.);; 2 Department of Paediatrics and Adolescent Medicine, School of Clinical Medicine, LKS Faculty of Medicine, The University of Hong Kong, Hong Kong SAR, China; 3 Neuro-Oncology Section, Hospital for Sick Children, Toronto, ON M5G1E8, Canada; 4 Department of Paediatrics, Prince of Wales Hospital, Hong Kong SAR, China; 5 Department of Paediatrics, Queen Elizabeth Hospital, Hong Kong SAR, China; 6 Department of Paediatrics, Faculty of Medicine, The Chinese University of Hong Kong, Hong Kong SAR, China; yukylngho001@cuhk.edu.hk; 7 Department of Medicine, United Christian Hospital, Hong Kong SAR, China; 8 Division of Nephrology, Department of Medicine and Geriatrics, Queen Mary Hospital, School of Clinical Medicine, LKS Faculty of Medicine, The University of Hong Kong, Hong Kong SAR, China; desmondy@hku.hk; 9 Paediatric Nephrology Centre, Department of Paediatrics and Adolescent Medicine, Hong Kong Children’s Hospital, Hong Kong SAR, China

**Keywords:** nephrotoxicity, osteosarcoma, survivorship, paediatric cancer

## Abstract

Background: The long-term kidney outcomes in paediatric osteosarcoma survivors treated with nephrotoxic chemotherapeutic agents are not well-described. Methods: We conducted a multi-centre retrospective cohort study and recruited paediatric osteosarcoma survivors who survived beyond 5 years from cancer diagnosis over a 20-year period. Chronic kidney disease (CKD) was defined as evidence of kidney impairment, chronic tubulopathy, and proteinuria upon last follow-up. Results: We included 89 patients (57% males) with a mean follow-up period of 14.9 years. A total of 42 subjects (47.2%) developed CKD, 28 of whom had CKD stage 1 with either chronic tubulopathy or proteinuria; 14 patients had CKD stage 2 (eGFR < 90 mL/min/1.73 m^2^), while two had CKD stage 3 (eGFR < 60 mL/min/1.73 m^2^). Chronic tubular dysfunction was reported in 34 patients (38%), with hypomagnesemia being the most common manifestation. Proteinuria and hypertension were infrequently observed, in 3% and 8% of cases, respectively. We found that a history of severe AKI and aminoglycoside exposure during the treatment course were significant risk factors for CKD, but the dose-dependent relationships between the development of CKD and cisplatin, ifosfamide, and methotrexate could not be demonstrated. Conclusions: CKD is prevalent among paediatric osteosarcoma survivors. Caution is needed when using multiple nephrotoxic agents at the same time, as this could increase the risk of CKD in the long run. Multi-disciplinary regular surveillance should be performed to identify and manage CKD early, including kidney function, electrolyte, and proteinuria monitoring.

## Simple Summary

This retrospective territory-wide cohort study examined long-term kidney outcomes in 89 paediatric osteosarcoma survivors (57% males, mean follow-up 14.9 years) treated with nephrotoxic agents like cisplatin, ifosfamide, and methotrexate, as well as aminoglycosides. Nearly half (47.2%) developed chronic kidney disease (CKD), primarily stage 1 (28 cases with tubulopathy or proteinuria), stage 2 (14 cases, eGFR < 90 mL/min/1.73 m^2^), or stage 3 (2 cases, eGFR < 60). Chronic tubulopathy affected 38%, with hypomagnesemia most common; proteinuria (3%) and hypertension (8%) were rare. History of severe AKI and aminoglycoside use were significant CKD risk factors, though dose relationships with key chemotherapies were unclear. Routine multidisciplinary surveillance is recommended to detect and manage CKD early.

## 1. Introduction

The advancement of cancer treatments has led to significant improvement in childhood cancer survival over the past several decades. Late complications secondary to treatment-related morbidities remain a concern among cancer survivors. One of the most well-established long-term morbidities is treatment-related nephrotoxicity, such as chronic kidney disease (CKD), with or without the need for kidney replacement therapy (KRT) [1,2]. CKD may manifest as impaired kidney function (estimated glomerular filtration rate, eGFR < 90 mL/min/1.73 m^2^). It may also present with normal kidney function, but other evidence of chronic kidney dysfunction, including tubulopathy and proteinuria. Childhood cancer survivors treated in the 1970s–1980s experienced 9 times greater risk of developing kidney failure than their siblings [3].

Contemporary treatment regimens have been refined to reduce potential nephrotoxicity. However, the mainstay treatments in most childhood cancer conditions still comprise nephrotoxic chemotherapies. In particular, for osteosarcoma, in addition to surgical resection of primary tumour, treatment traditionally involves chemotherapy with cisplatin, high-dose methotrexate, and ifosfamide. These agents render osteosarcoma patients more susceptible to developing CKD than other childhood cancer survivors. A recent study showed the odds ratio of developing CKD stage 2 or above was 4.67 in osteosarcoma survivors, compared to those with hepatoblastoma [4]. In the Childhood Cancer Survivor Study, osteosarcoma survivors were found to be the second most at-risk population after Wilms’ tumour patients for developing late-onset kidney failure [5]. Most long-term survivorship studies have focused on measuring the decrease in GFR. While short-term outcomes, such as acute kidney injury (AKI), as well as tubular dysfunction, resulting from cisplatin, methotrexate, and ifosfamide have been well-reported [6,7,8], few studies have evaluated the more nuanced or specific aspects of the long-term impacts, such as chronic tubulopathy. Further, exposure to other concurrent medications, including broad-spectrum antibiotics and anti-fungal agents, may result in further cumulative injuries to the nephrons, with limited data in the literature.

In view of current gaps in knowledge pertaining to the long-term kidney sequelae among paediatric osteosarcoma survivors, the aim of this multi-centre study was to examine kidney outcomes, including chronic kidney disease and tubulopathy, over a 20-year period and to identify potential associated factors to improve patient outcomes.

## 2. Methods

We conducted a multi-centre retrospective observational cohort study on biopsy-proven childhood osteosarcoma survivors diagnosed before the age of 19. We included all long-term survivors, defined as patient survival for five or more years after initial diagnosis. Patients included were treated and/or being followed at four tertiary referral centres, including Hong Kong Children’s Hospital, Queen Mary Hospital, Prince of Wales Hospital and Queen Elizabeth Hospital over a 20-year period between from 1 January 1999 to 31 December 2018. Patients who had alternative diagnoses, died, or were lost to follow-up in fewer than 5 years from diagnosis, and those with incomplete treatment data, were excluded.

Patients were identified from an existing patient registry of the Hong Kong Paediatric Haematology Oncology Study Group (HKPHOSG). Patients were followed in the cancer survivor clinics of their respective hospitals annually, with blood pressure measurement, blood and urinary tests. Anonymised data on patient demographics, medications, radiological results and laboratory parameters were collected through the Clinical Management System (CMS) of Hong Kong Hospital Authority.

**Figure 1:**
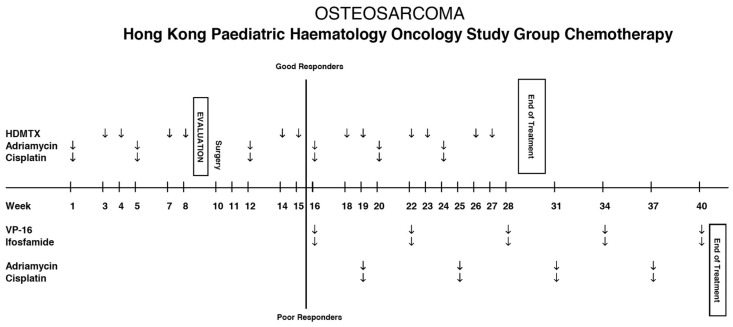
Roadmap of the Hong Kong Paediatric Haematology Oncology Study Group (HKPHOSG) treatment protocol for paediatric osteosarcoma (HDMTX = high-dose methotrexate; VP-16 = etoposide).

All osteosarcoma patients were treated with the standard HKPHOSG protocol, as illustrated in Figure 1. The HKPHOSG protocol comprised methotrexate, Adriamycin, and cisplatin (MAP), with or without intensified chemotherapy including ifosfamide and etoposide (MAPIE). These agents are commonly used in other adult and paediatric osteosarcoma protocols, including the most-referred European and American Osteosarcoma Study Group (EURAMOS) protocol in the field [9]. During the initial 15 weeks of treatment, one cycle of doxorubicin and cisplatin was given after every two cycles of high-dose methotrexate (HD-MTX). Evaluation imaging, followed by surgical resection, were performed after eight weeks of neoadjuvant chemotherapy. All cases continued with the same regimen as adjuvant chemotherapy until week 16. At week 16, subjects were stratified into two treatment arms based on the pathological response of the resected tumour. Patients with >90% tumour necrosis (<10% viable tumour) on histology were classified as ‘good responders’ according to standard terminology. They continued to receive the same chemotherapy until week 27. On the other hand, subjects with <90% tumour necrosis (≥10% viable tumour) were defined as ‘poor responders’ and would be given cycles of VP-16 and ifosfamide (instead of HD-MTX as in ‘good responders’), alternating with cycles of doxorubicin and cisplatin from weeks 16 to 34. Table 1 summarises the total dosage of chemotherapy given in the ‘good’ and ‘poor responders’ subgroups.

Primary outcome was CKD, which included the development of impaired kidney function (eGFR < 90 mL/min/1.73 m^2^), chronic tubular dysfunction, and proteinuria. CKD staging was defined according to the eGFR according to the Kidney Disease: Improving Global Outcomes Organization [KDIGO] 2024 clinical practice guideline [10]. Patients with normal kidney function (eGFR > 90 mL/min/1.73 m^2^), who had tubular loss of electrolytes and/or proteinuria, were defined as having CKD stage 1. Evidence of chronic kidney dysfunction should last for 3 months or more. eGFR was calculated by serum creatinine using the full-age spectrum (FAS) equation, a single formula that could be applied to both paediatric and adult populations and minimised the need to switch between age-specific equations [11]. The FAS equation has been validated in various patient populations, including healthy Chinese individuals, Chinese adults with CKD, and children with cancers [12,13,14,15]. Chronic tubulopathy was defined as either the need for long-term electrolyte supplementation (>1 type) or an abnormally low serum level of potassium, phosphate, magnesium, or bicarbonate at last follow-up. Proteinuria was quantified by urinary protein-to-creatinine ratio (UPCR) collected from the first void urine in the morning. Hypertension was defined as an elevated systolic and/or diastolic blood pressure that was >95th percentile for their age, sex and height on three visits or more. Severe stage 3 acute kidney injury (AKI) was defined by a three-fold increase in serum creatinine [16]. Complete remission (or relapse-free) was defined as no detectable disease upon imaging studies of the primary site, without evidence of malignancy on bone scan and computer tomography of thorax.

Statistical analysis was conducted using IBM SPSS version 27 and R version 4.5.2 with “stats” and “car” packages. Descriptive statistics, including mean, standard deviation (SD), median, interquartile range (IQR), count, and percentage (%), were applied as appropriate. Potential factors were compared to identify associated factors for developing CKD, using Mann–Whitney U test for continuous variables and Fisher’s exact test for categorical variables, as appropriate. Additionally, a multiple logistic regression model was adopted to determine the relationship between CKD and the potential risk factors. These factors included age at first cancer diagnosis, sex, metastatic site, treatment group, use of nephrotoxic antimicrobials (namely vancomycin, amphotericin B, and aminoglycosides), history of severe AKI, history of nephrocalcinosis, and disease recurrence. Multivariable analysis included only covariates with a *p*-value < 0.1 in the univariable models. Multicollinearity in the model was checked using variance inflation factor. Kaplan–Meier analysis was used to illustrate the development of CKD (stage 2 or above) or kidney failure from initial cancer diagnosis. Patients were censored at last follow-up. For all statistical tests, *p*-value < 0.05 would be treated as significant.

## 3. Results

### 3.1. Patient Characteristics

Over the 20-year study period, we identified 93 children diagnosed with biopsy-proven osteosarcoma who survived for 5 or more years following initial diagnosis. After excluding four cases with incomplete treatment or follow-up data, a total of 89 patients (57.3% male; mean age at diagnosis, 13.2 ± 3.51 years) were included in the final analysis. Detailed patient demographics are shown in Table 2.

### 3.2. Treatment Details and Response

All patients received the aforementioned standard treatments. Forty-six patients (49.5%) showed >90% tumour necrosis following neoadjuvant chemotherapy, defined as good responders, and continued with the initial treatment. Forty-three children were poor responders, with <90% tumour necrosis, and were treated with additional etoposide and ifosfamide and reduced doses of cisplatin and methotrexate. Chemotherapy dosing was adjusted in four children due to treatment-related toxicity, with details shown in Table 3.

Most osteosarcoma survivors (*n* = 78/89, 87.6%) remained in complete remission (CR1). Eleven subjects (12.4%) relapsed after first remission. Of these patients, 10 achieved second complete remission (CR2). Four required surgical resections of lung metastasis, and six received second-line chemotherapy. The chemotherapy regimens included high-dose ifosfamide (15 g/m^2^/course for 6–12 courses), ifosfamide & carboplatin & etoposide (ICE) regimen, or gemcitabine & doxorubicin (GEMDOX), with or without tyrosine kinase inhibitors (apatinib). The remaining patient suffered from isolated pulmonary relapse for a second time but also achieved remission (CR3) after complete surgical resection, without adjuvant chemotherapy.

One patient died 9 years after initial cancer diagnosis. The patient had underlying neurofibromatosis type 1 and developed a second cancer of malignant neural sheath tumour with distant metastasis.

### 3.3. Renal Outcomes

#### 3.3.1. Kidney Impairment and Chronic Kidney Disease

**Figure 2:**
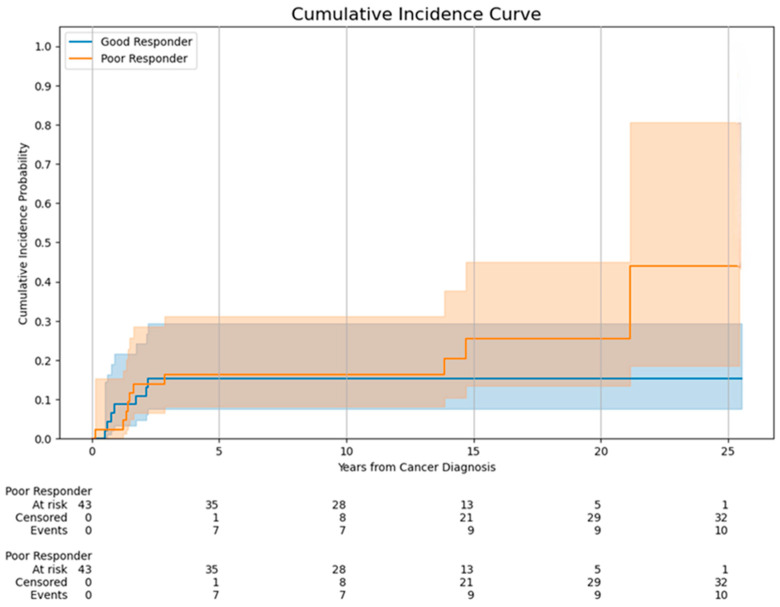
Incidence of stage 2 or above CKD (eGFR < 90 mL/min/m^2^) in childhood osteosarcoma survivors.

At last follow-up, the median serum creatinine and eGFR of all long-term survivors were 78 µmol/L (IQR, 60.0–90.3) and 103.4 mL/min/1.73 m^2^ (IQR, 88.7–118.3), respectively (Table 4). Of the 89 subjects, 42 patients (47.2%) developed CKD at last follow-up. Poor responders demonstrated a trend of higher CKD incidence compared to good responders (53.5% vs. 41.3%, *p* = 0.29). Majority of patients (*n* = 28/42, 66.7%) developing CKD manifested either proteinuria and/or tubular dysfunction with normal kidney function (stage 1). Fourteen patients developed kidney impairment (CKD stage 2 or above), with a median time of 1.6 years (IQR, 1.3–2.0 years) from first cancer diagnosis. Two patients had CKD stage 3, and none developed stage 4 or 5 CKD. Ten out of these 14 patients with kidney impairment (70%) were ‘poor responders’ (*p* = 0.08). Kaplan–Meier analysis showed a higher incidence of CKD stage 2 or above among poor responders, although log rank test showed *p* = 0.22 (Figure 2).

#### 3.3.2. Tubular Dysfunction

Thirty-four patients (38.2%) demonstrated evidence of chronic tubular dysfunction, with similar prevalence rates between the two responder groups. Among the 34 patients with chronic tubular dysfunction, 22 (64.7%), 15 (44.1%) and 16 (47.1%) had hypomagnesaemia, hypophosphatemia and/or hypokalaemia, respectively. Fifteen (44.1%) patients received one or more types of long-term electrolyte replacement at the last follow-up. No patients had metabolic acidosis or required sodium bicarbonate supplementation. One subject in our cohort developed severe Fanconi syndrome, which required changing chemotherapy from ifosfamide (15 g/m^2^) to cyclophosphamide (2.5 g/m^2^). She eventually progressed to stage 2 CKD. At last follow-up, she did not require regular phosphate and magnesium supplementation. Ultrasound of urinary system also showed early nephrocalcinosis. The detailed summary is described in Table 3, above.

#### 3.3.3. Proteinuria and Hypertension

Proteinuria was detected in three patients at last follow-up (CKD stage 1, *n* = 2; CKD stage 2, *n* = 1), with UPCR ranging from 0.22 to 0.5 mg/mg. None of these patients received renin–angiotensin–aldosterone system inhibitor (RAASi). The serum albumin levels of all patients were normal at last follow-up. Seven patients were confirmed to have hypertension. Two of them had stage 2 hypertension. Details of renal outcomes are summarised in Table 4, above.

### 3.4. Risk Factors for Nephrotoxicity

We analysed potential factors associated with CKD, including sex, age at diagnosis, year of treatment, disease recurrence, metastatic disease, history of AKI, evidence of nephrocalcinosis, and use of nephrotoxic medications during cancer treatment period in Table 5. Almost 60% of patients received one or more courses of nephrotoxic antimicrobials. These included vancomycin (*n* = 28, 31.5%), aminoglycoside (*n* = 34, 38.2%) and amphotericin B (*n* = 2, 2.2%). There is no significant difference in the proportions of patients exposed to nephrotoxic antimicrobials between the good and poor responder treatment groups. Exposure to aminoglycosides was significantly associated with CKD when compared with no exposure, especially pertaining to chronic tubulopathy (*p* = 0.01). Multivariable analysis showed the odds ratio for developing CKD in those exposed to aminoglycosides increased to 3.19 (95% confidence interval: 1.29, 8.23; *p* = 0.01).

Severe AKI during cancer treatment was documented in 11 patients (12.4%). The cause of AKI was multi-factorial, secondary to chemotherapy and concurrent infection. None of them required acute kidney support, and all their kidney function improved after fluid replenishment and treating infections. Odds ratio for developing CKD by multivariable analysis was significantly increased to 4.79 in those with history of severe AKI (95% confidence interval: 1.21, 24.3; *p* = 0.03). Two patients (2.2%) were incidentally found to have nephrocalcinosis on imaging. However, it was not shown to be associated with CKD development. Finally, no correlations with CKD were observed with the dosing of cisplatin, ifosfamide, or methotrexate. Other detailed risk factor analysis can be found in Table 6.

## 4. Discussion

In this territory-wide multi-centre cohort study of paediatric osteosarcoma survivors, we provided long-term, osteosarcoma-specific data on both glomerular and tubular kidney outcomes over a mean follow-up approaching 15 years. Our focus on systematic assessment of electrolyte-wasting tubulopathy, alongside eGFR-based impairment, reveals a substantial burden of subclinical CKD that would be under-recognised by eGFR alone. This study, therefore, complements and extends prior survivorship cohorts that either included heterogeneous cancer types or emphasised reduced eGFR without tubulopathy data [9,17,18].

We observed that almost half of survivors (47%) developed some degree of chronic kidney dysfunction years from cancer diagnosis. While about 16% of patients had impaired kidney function, nearly one-third of our cases demonstrated chronic tubulopathy, requiring long-term electrolyte supplementation. As previously mentioned, although there are differences in cumulative dosages of cisplatin and ifosfamide, our findings can be generalised to a large proportion of paediatric and young adult cohorts worldwide using similar chemotherapy regimens. In 2016, a study from the EURAMOS showed that the IE regimen had a comparable 3-year event-free survival to conventional regimen of using only MAP, but was associated with increased toxicities [19]. Our group, therefore, stopped using IE-containing regimen in newly diagnosed osteosarcoma since 2020, in order to reduce nephrotoxicity. This change in treatment protocol, however, did not affect our findings, since the last enrolled patient was treated in 2018.

The incidence of CKD among childhood cancer survivors ranges from 2.4% to 32%, depending on the study design, cancer types, follow-up duration, and definitions of nephrotoxicity [1,2,5,20,21,22,23,24,25]. Many of these studies include various cancer types, including sarcomas or Wilms’ tumour, where radiation therapy to abdomen and/or nephrectomy could be contributing significantly to the kidney outcomes. Much focus is put on measurement of renal function by eGFR-Cr, whereas tubular abnormalities are often underreported. Even large cohorts, including St. Jude’s Lifetime Cohort Study, the 7.7% (9 out of 117) osteosarcoma survivors (in their subgroup analysis) who developed CKD were defined by impaired kidney function using eGFR < 90 mL/min/1.73 m^2^) [26]. They did not systematically capture electrolyte-wasting or protein-losing states, either.

Furthermore, some paediatric cancer survivorship studies investigated the CKD prevalence merely through self-reported questionnaires [5,26] or diagnostic coding [27]. No data pertaining to tubular dysfunction, proteinuria, and hypertension were reported, which should be considered as manifestations of CKD even without kidney function impairment. Consequently, epidemiology of CKD, especially for patients with isolated tubulopathy, is likely to be under-reported in the current literature. Indeed, we reported a higher incidence of CKD, mostly due to a high incidence of chronic tubulopathy (38%) secondary to magnesium, phosphate and/or potassium urinary wasting, without reduction in eGFR. Our extended follow-up period of 15 years also enables accurate evaluation of these late effects, which may only manifest in later life.

It is reported that the incidence of nephrotoxicity is much higher, at over one-fifth to half of adult osteosarcoma patients [28,29]. In contrast, our cohort comprises adolescents and young adults who are long-term survivors of paediatric osteosarcoma, among whom most kidney abnormalities are early-stage. The higher rates and severity of nephrotoxicity described in adult cohorts likely reflect differences in chemotherapy delivery and baseline vulnerability: adults often receive higher absolute doses, with reduced physiological renal reserve—particularly those aged ≥50 years, coinciding with the second incidence peak of osteosarcoma—and carry a greater burden of comorbidities (e.g., hypertension, diabetes, atherosclerosis) that amplify nephrotoxic risk. Survivorship trajectories, therefore, differ substantially, and adult data should only be extrapolated to paediatric practice with caution. While adult patients face lower long-term survival with competing cardiovascular risks, paediatric survivors have a prolonged lifespan, during which subclinical tubular injury—even if initially mild—may persist, accrue, or progress. Accordingly, the increased lifetime risk of progression from stage 1 to 2 CKD toward kidney failure in paediatric survivors warrants proactive management and patient education to avoid additional nephrotoxic exposures from lifestyle and medications.

Regarding specific contributing factors on kidney outcomes, cisplatin and ifosfamide are well-documented nephrotoxic agents that can cause acute glomerular damage and even severe AKI. It is reported that partial recovery can predispose survivors to develop CKD [27] In our cohort, a history of severe AKI increased the odds of developing CKD by almost 5-fold. This suggests that, although AKI episodes might be transient and reversible, severe AKI could still predispose to the development of CKD. This highlights the clinical challenge of balancing adequate oncological dosing with renal safety. Rather than empirical dose reductions, strategies such as aggressive hydration, avoidance or minimisation of concurrent nephrotoxic medications, timely treatment of sepsis, and close pharmacokinetic monitoring (particularly for high-dose methotrexate) may allow delivery of curative-intent chemotherapy. while limiting kidney injury. Early nephrology involvement during episodes of AKI may further facilitate recovery and guide safe continuation of therapy without compromising cancer outcomes.

The Childhood Cancer Survivor Study in the United Kingdom reported chronic glomerular nephrotoxicity was present in up to 50% survivors treated with ifosfamide and 80% of those treated with cisplatin [24,30]. Our study, however, did not identify any dose-dependent relationship between these two agents and CKD. This concurs with findings from other long-term follow-up studies [6,27,31]. Methotrexate is another medication which can cause acute, reversible kidney impairment. The risk can be mitigated through hyperhydration, urine alkalinisation protocols, folinic acid rescue, and close monitoring of drug clearance [8]. Our findings also failed to demonstrate an association between high-dose methotrexate and CKD. This conclusion, however, has to be interpreted with caution due to the small sample size.

Tubular dysfunction is commonly observed during chemotherapy [1,7], which may spontaneously recover in a subset of patients [1,20,31]. In a small number of patients, change in chemotherapy regimen is required in view of severe tubulopathy. In our study, more than half of the patients developed chronic tubulopathy, and nearly one-third required long-term electrolyte supplementation. Hypomagnesaemia was the most common manifestation, which could be attributed to cisplatin. Cisplatin can also result in secondary hypocalcaemia [24,32]. Ifosfamide is associated with generalised proximal tubular loss and Fanconi syndrome [2,7,31]. In contrast, methotrexate is not associated with tubulopathy. Although tubular toxicity due to ifosfamide has been reported to improve with time [33], hypomagnesemia resulting from cisplatin persists in 10–30% of cases [27,30]. Indeed, a significant proportion of survivors remained on phosphate and magnesium supplementation even years after chemotherapy exposure. We did not observe a dose-dependent relationship between ifosfamide and cisplatin and chronic tubulopathy. This is contrary to previous findings that higher ifosfamide dosing was associated with proximal tubulopathy [34,35], and platinum agents could lead to acute and chronic glomerular and tubular damage in 60% of children [24].

About 3% and 8% of our patients developed proteinuria and hypertension, which can occur as isolated features or part of the CKD manifestations. Their presence also accelerates CKD progression. Hypertension is associated with ifosfamide and cisplatin use, even without documented glomerular damage [34]. Compared to our osteosarcoma survivors, previous studies reported higher rates of proteinuria and hypertension among paediatric patients surviving Wilms’ tumour or abdominal sarcomas, which could be a result of chronic radiation nephropathy and/or glomerular hyperfiltration post-nephrectomy [2,3,21,24]. In osteosarcoma, radiation therapy is rarely required, which may account for the lower rates of proteinuria and hypertension in our cohort. Tyrosine kinase inhibitors (TKIs), such as apatinib and lenvatinib, are increasingly used in refractory or relapsing paediatric osteosarcoma and can cause proteinuria and hypertension [36,37]. The drug was only used in two of our relapsed patients, but neither experienced chronic kidney impairment, proteinuria, or hypertension. Larger-scale long-term safety data is warranted, and regular surveillance should be offered to patients receiving tyrosine kinase inhibitors. Reno-protective role of RAASi is well-reported in both adults and children [38,39,40]. With earlier detection, it can be prescribed to slow down the progression of chronic kidney disease. However, the number of patients with proteinuria and hypertension in our cohort was small, and none were receiving renin–angiotensin–aldosterone system inhibitors at last follow-up. We were, therefore, unable to assess the reno-protective impact of RAAS inhibition in this population. Some newer reno-protective options, such as sodium-glucose cotransporter, should be investigated in this specific patient population, as well [41].

Similarly, electrolyte supplementation—most commonly magnesium and phosphate—was required in a substantial proportion of patients with chronic tubulopathy, as mentioned above, which can lead to biochemical stabilisation, but not necessarily reversal of tubular dysfunction. These observations underscore the importance of early detection, as interventions at a subclinical stage may help mitigate downstream CKD progression.

Apart from chemotherapy, exposure to nephrotoxic antimicrobial is a frequent cause of AKI during cancer treatment. This has not been identified to be a significant predictor for CKD in other paediatric survivorship cohorts [21,42]. Nonetheless, aminoglycoside can cause acute proximal tubular injury, and it is plausible that the drug, with repeated or cumulative use, may lead to chronic kidney dysfunction [43,44]. In this context, our data demonstrated a positive association between cumulative aminoglycoside exposure and CKD, which was further confirmed upon multivariable analysis. This highlights the importance of limiting nephrotoxic agent use through close surveillance and clinical pharmacist support, especially among this high-risk patient population.

Our study has several limitations. First, our study was retrospective in nature, subject to potential selection and reporting bias. Patients who did not fulfil the criteria for long-term survival (>5 years) or were lost to follow-up might have had CKD but were not included. Second, although we had a relatively large paediatric cohort, the sample size remained small, which limited the statistical power in our analyses. Third, the lack of histological changes that lead to CKD cannot be ascertained, since kidney biopsy was not performed in most cases. Last, laboratory data prior to the year 2000 was incomplete, and there was a lack of standardised protocol on monitoring of electrolytes, proteinuria, and kidney function, which may have influenced the detection of these complications. Future prospective multi-centre studies employing more precise methods for renal function assessment are warranted to clarify risk factors and dose thresholds.

In summary, nephrotoxicity is common in paediatric osteosarcoma patients. Hence, kidney function, electrolyte and acid–base balance, proteinuria, and blood pressure evaluations should be considered as integral to regular surveillance among paediatric osteosarcoma survivors. Preferably, paediatric or adult nephrologists should be actively involved in the survivorship programme. A multidisciplinary team approach will enable early detection of AKI and CKD, and timely intervention to limit comorbidities. We suggest at least annual assessment of the following: (i) kidney function (serum creatinine with eGFR calculation), (ii) blood pressure, (iii) urine protein excretion (first-morning UPCR when feasible), and (iv) serum electrolytes with emphasis on magnesium, phosphate, and potassium. More frequent monitoring (e.g., every 3–6 months in the first 2–3 years after therapy completion) may be warranted for patients with documented tubular dysfunction, poor chemotherapy response, or substantial exposure to nephrotoxic antimicrobials. Lifelong follow-up should be considered in individuals with persistent electrolyte wasting, even when eGFR remains >90 mL/min/1.73 m^2^. With the implementation of a well-structured, multi-disciplinary, long-term follow-up monitoring programme, future prospective multi-centre studies on developing less nephrotoxic treatment regimens and identifying more precise biomarkers that can predict CKD development would be the next step forward. Additionally, the impact of using novel agents such as TKIs, and those relapsed/refractory patients receiving multiple lines of treatment regimens, could be studied.

## 5. Conclusions

CKD is prevalent among paediatric osteosarcoma survivors, affecting nearly half of our territory-wide cohort with a mean follow-up of 14.9 years. While 16% of the survivors demonstrated impaired kidney function, chronic tubulopathy was observed in 38%—most commonly hypomagnesemia requiring long-term electrolyte supplementation. Aminoglycoside exposure and history of severe AKI were significant risk factors for CKD development.

Ultimately, recognising CKD in paediatric osteosarcoma survivors as a spectrum—including glomerular, tubular, and hypertensive manifestations—provides an opportunity for earlier intervention and long-term preservation of kidney health without compromising oncological cure. Comprehensive surveillance and multidisciplinary collaboration with nephrologists would facilitate early detection and intervention. Future studies should explore nephrotoxicity-sparing regimens and reno-protective strategies for this high-risk survivor population.

## Figures and Tables

**Table 1:** Cumulative chemotherapy dosages described in the HKPHOSG paediatric osteosarcoma protocol.

Chemotherapeutic Agent	Good Responders	Poor Responders
Cisplatin	600 mg/m^2^	700 mg/m^2^
High-dose methotrexate (HD-MTX)	144 g/m^2^	72 g/m^2^
Ifosfamide	Nil	37.5 g/m^2^
Etoposide (VP-16)	Nil	1500 mg/m^2^
Doxorubicin	360 mg/m^2^	420 mg/m^2^

**Table 2:** Demographics of all 89 paediatric osteosarcoma survivors.

	Overall, *n =* 89		Good Responders ^1^, *n* = 46		Poor Responders ^2^, *n* = 43	
Sex: male, *n* (%)	51	57.3%	25	54.3%	16	37.2%
Age at cancer diagnosis, mean (SD)	13.2	(3.51)	13.0	(3.38)	13.4	(3.67)
Age at last follow-up, mean (SD)	28.1	(6.09)	28.7	(6.04)	27.4	(6.14)
Time of last follow-up from cancer diagnosis (in years), mean (SD)	14.9	(5.75)	15.7	(6.14)	14.1	(5.23)
Primary site, *n* (%)						
Proximal tibia	21	23.6%	12	26.1%	9	20.9%
Distal femur	49	55.1%	25	54.3%	24	55.8%
Proximal fibula	7	7.9%	3	6.5%	4	9.3%
Humerus	8	9.0%	4	8.7%	4	9.3%
Distal radius	1	1.1%	1	2.2%	0	0.0%
Metastatic diseases, *n* (%)	18	20.2%	7	15.2%	11	25.6%
Lung	17	19.1%	7	15.2%	10	23.3%
Bone	1	1.1%	0	0.0%	1	2.3%
Serum creatinine at diagnosis, umol/L, mean (SD)	56.4	(14.54)	54.3	(12.02)	58.6	(16.60)
eGFR (by FAS) at diagnosis, ml/min/1.73 m^2^, mean (SD)	104.5	(20.77)	105.6	(24.35)	103.5	(16.57)
Death (beyond 5-year survival), *n* (%)	1	1.1%	1	2.2%	0	0.0%

^1^ ‘Good responders’ refer to the group of osteosarcoma patients with >90% tumour necrosis, who received methotrexate, Adriamycin and platinum (Cisplatin) [MAP]. ^2^ ‘Poor responders ‘refer to the group of osteosarcoma patients with <90% tumour necrosis, who received intensified chemotherapy of methotrexate, Adriamycin and platinum (Cisplatin) alternating with ifosfamide and etoposide [MAPIE].

**Table 3:** Summary on cases with chemotherapy dosage adjustment at physician’s discretion.

Case No.	Disease Details	Initial Treatment Arm	Modification(s)	Reason(s)	Disease Status	Renal Outcome
62	Localised disease at right proximal tibia	Poor responder	Omitted MTX from week 4 onwards (Total MTX dose = 24 g/m^2^)	Neurotoxicity	CR1	Stage 1 CKD (normal eGFR but tubular dysfunction)
66	Localised disease at left distal femur	Good responder	To poor responder arm, with 2/3 dosage since week 12 (Total cisplatin dose = 275 mg/m^2^; Total MTX dose = 48 g/m^2^; Total ifosfamide dose = 17 g/m^2^)	Profound marrow suppression	CR3	No kidney dysfunction
89	Localised disease at right distal femur	Poor responder	1. Replaced ifosfamide with CTX since week 22 2. 50% reduction in MTX since week 8 with aminophylline prophylaxis (Total cisplatin dose = 500 mg/m^2^; Total MTX dose = 42 g/m^2^; Total CTX dose = 2.5 g/m^2^; Total ifosfamide dose = 15 g/m^2^)	1. Severe Fanconi syndrome since week 22 2. MTX leuko-encephalopathy in week 7	CR1	Stage 2 CKD
90	Localised disease at left proximal tibia	Good responder	Changed to poor responder arm after week 14 (Total cisplatin dose = 400 mg/m^2^; Total MTX dose = 60 g/m^2^; Total ifosfamide dose, including relapse = 217.5 g/m^2^)	Allergic reaction to HD-MTX at week 14	CR2	No kidney dysfunction

CKD = chronic kidney disease; CR = complete remission; CTX = cyclophosphamide; MTX = methotrexate.

**Table 4:** Renal outcomes in paediatric osteosarcoma survivors.

	Overall, *n* = 89		Good Responders, *n* = 46		Poor Responders, *n* = 43		*p*-Value
Any stage of CKD, *n* (%)	42	(47.2%)	19	(41.3%)	23	(53.5%)	0.29
Stage 1 CKD, *n* (%)	28	(31.5%)	15	(32.6%)	13	(30.2%)	0.82
Stage 2 CKD, *n* (%)	14	(15.7%)	4	(8.7%)	10	(23.8%)	0.08
Time to reach stage 2 CKD (in years), median (IQR)	1.6	(1.3–2.0)	0.8	(0.6–1.7)	1.5	(1.3–13.8)	0.13
Stage 3 CKD, *n* (%)	2	(2.2%)	1	(2.2%)	1	(2.3%)	1.00
Time to reach stage 3 CKD (in years), median (range)	7.1	(1.1–13.1)	1.1	-	13.1	-	N/A
Serum creatinine at last follow-up, umol/L, median (IQR)	78.0	(60.0–90.3)	73.0	(56.0–86.0)	81.0	(69.0–94.0)	0.75
eGFR at last follow-up, ml/min/1.73 m^2^, median (IQR)	103.4	(88.7–118.3)	105.8	(92.3–132.1)	100.3	(88.5–107.3)	1.00
Evidence of chronic tubulopathy, *n* (%)	34	(38.2%)	18	(39.1%)	16	(37.2%)	1.00
Magnesium tubular loss	22	(24.7%)	13	(28.3%)	9	(21.4%)	0.47
Phosphate tubular loss	15	(16.9%)	7	(15.2%)	8	(18.6%)	0.78
Potassium tubular loss	16	(18.0%)	10	(21.7%)	6	(14.0%)	0.41
Proteinuria, *n* (%)	3	(3.4%)	1	(2.2%)	2	(4.7%)	0.61
Hypertension, *n* (%)	7	(7.9%)	6	(13.0%)	1	(2.3%)	0.11
Death, *n* (%)	1	(1.1%)	1	(2.2%)	0	(0.0%)	1.00

**Table 5:** Factors associated with chronic kidney disease.

	Overall, *n* = 89		Any Stage of CKD, *n* = 42			Chronic Tubulopathy, *n* = 34			Proteinuria, *n* = 3		
	*n*	%	*n*		*p*-Value	*n*		*p*-Value	*n*		*p*-Value
Poor treatment response	49	55.1%	27	64.3%	0.52	18	52.9%	0.83	3	100%	0.25
History of severe AKI	11	12.4%	8	19.0%	0.11	3	8.8%	0.52	0	0.0%	1.00
History of nephrocalcinosis	2	2.2%	2	4.8%	0.22	2	5.9%	0.14	1	33.3%	0.0023
Use of nephrotoxic antimicrobials	50	56.2%	27	64.3%	0.20	25	73.5%	0.0151	2	66.7%	1.00
Vancomycin	28	31.5%	16	38.1%	0.26	15	44.1%	0.0602	2	66.7%	0.23
Amphotericin B	2	2.2%	1	2.4%	1.00	1	2.9%	1.00	0	0.0%	1.00
Aminoglycosides	34	38.2%	16	38.1%	0.049	19	55.9%	0.013	2	66.7%	0.56
Disease recurrence	11	12.4%	6	14.3%	0.75	6	17.6%	0.32	2	66.7%	0.039

**Table 6:** Risk factor analysis for chronic kidney disease.

	Univariable			Multivariable		
	ORunadj	(95% CI)	*p*-Value	ORadj	(95% CI)	*p*-Value
Sex						
Female	1	[Ref]				
Male	0.82	(0.35, 1.91)	0.65			
Age at first cancer diagnosis	1.00	(0.88, 1.12)	0.96			
Year of treatment	1.02	(0.95, 1.11)	0.56			
Stage of disease						
Localised disease	1	[Ref]				
Lung metastasis	1.30	(0.45, 3.82)	0.63			
Bone metastasis	0.00	(NA, NA)	0.99			
Use of ifosfamide	1.63	(0.71, 3.82)	0.25			
History of severe AKI	3.45	(0.92, 16.70)	0.08	4.79	(1.21, 24.3)	0.03
History of nephrocalcinosis	0.00	(NA, NA)	0.99			
Use of vancomycin	1.79	(0.73, 4.51)	0.21			
Use of amphotericin B	1.12	(0.04, 29.00)	0.94			
Use of aminoglycosides	2.62	(1.10, 6.44)	0.03	3.19	(1.29, 8.23)	0.01
Disease recurrence						
CR1	1	[Ref]				
CR2/CR3	1.40	(0.39, 5.22)	0.6			

## Data Availability

The raw data supporting the conclusions of this article is contained within the article. Further inquiries can be directed to the corresponding authors.

## Abbreviations

The following abbreviations are used in this manuscript:

AKI	Acute kidney injury
CKD	Chronic kidney disease
CMS	Clinical management system
CR	Complete remission
CTX	Cyclophosphamide
eGFR	Estimated glomerular filtration rate
EURAMOS	European and American Osteosarcoma Study
FAS	Full-age spectrum
GEMDOX	Gemcitabine–docetaxel
HD	High dose
HKPHOSG	Hong Kong Paediatric Haematology-Oncology Study Group
IE	Ifosfamide and etoposide
ICE	Ifosfamide–carboplatin–etoposide
IQR	Inter-quartile range
KDIGO	The Kidney Disease: Improving Global Outcomes Organization
KRT	Kidney replacement therapy
MAP	Methotrexate, Adriamycin, and Cisplatin
MTX	Methotrexate
SD	Standard deviation
TKIs	Tyrosine kinase inhibitors
UPCR	Urine protein-to-creatinine ratio
